# Role of the Skp1 prolyl-hydroxylation/glycosylation pathway in oxygen dependent submerged development of *Dictyostelium*

**DOI:** 10.1186/1471-213X-12-31

**Published:** 2012-10-25

**Authors:** Yuechi Xu, Zhuo A Wang, Rebekah S Green, Christopher M West

**Affiliations:** 1 Department of Biochemistry and Molecular Biology, Oklahoma Center for Medical Glycobiology, University of Oklahoma Health Sciences Center, 975 NE 10th St., BRC 413, OUHSC, Oklahoma City, OK, 73104, USA; 2 current address: Department of Molecular Microbiology, Washington University Medical School Campus, Box 8230, 660 South Euclid Avenue, St. Louis, MO, 63110, USA

**Keywords:** Prolyl 4-hydroxylase, Glycosyltransferase, Oxygen sensing, Hypoxia, Hydroxyproline, Cellular slime mold

## Abstract

**Background:**

Oxygen sensing is a near universal signaling modality that, in eukaryotes ranging from protists such as *Dictyostelium* and *Toxoplasma* to humans, involves a cytoplasmic prolyl 4-hydroxylase that utilizes oxygen and α-ketoglutarate as potentially rate-limiting substrates. A divergence between the animal and protist mechanisms is the enzymatic target: the animal transcriptional factor subunit hypoxia inducible factor-α whose hydroxylation results in its poly-ubiquitination and proteasomal degradation, and the protist E3^SCF^ubiquitin ligase subunit Skp1 whose hydroxylation might control the stability of other proteins. In *Dictyostelium*, genetic studies show that hydroxylation of Skp1 by PhyA, and subsequent glycosylation of the hydroxyproline, is required for normal oxygen sensing during multicellular development at an air/water interface. Because it has been difficult to detect an effect of hypoxia on Skp1 hydroxylation itself, the role of Skp1 modification was investigated in a submerged model of *Dictyostelium* development dependent on atmospheric hyperoxia.

**Results:**

In static isotropic conditions beneath 70-100% atmospheric oxygen, amoebae formed radially symmetrical cyst-like aggregates consisting of a core of spores and undifferentiated cells surrounded by a cortex of stalk cells. Analysis of mutants showed that cyst formation was inhibited by high Skp1 levels via a hydroxylation-dependent mechanism, and spore differentiation required core glycosylation of Skp1 by a mechanism that could be bypassed by excess Skp1. Failure of spores to differentiate at lower oxygen correlated qualitatively with reduced Skp1 hydroxylation.

**Conclusion:**

We propose that, in the physiological range, oxygen or downstream metabolic effectors control the timing of developmental progression via activation of newly synthesized Skp1.

## Background

**Figure 1:**
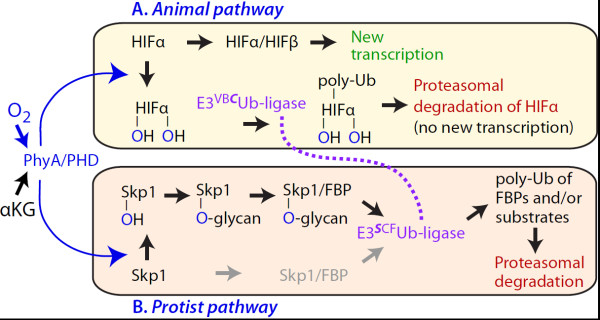
**Schematic comparison of O**_**2 **_**and prolyl 4-hydroxylase signaling in animals and protists.** (**A**) The upper panel shows current thinking about how O_2_- and α-ketoglutarate (αKG)-dependent hydroxylation of 2 Pro residues of HIFα by PHD2 generates a degron recognized by E3^VBC^Ub-ligase leading to its poly-ubiquitination and degradation in the 26S-proteasome, thereby interfering with its heterodimerization with HIFβ and induction of genes appropriate to response to low O_2_[1,2]. (**B**) The lower panel shows current thinking [11] about how the protist ortholog PhyA leads to hydroxylation and multi-step glycosylation of Pro143 (in *Dictyostelium*). Hydroxylation of Skp1 does not generate a degron [5], but it and glycosylation may affect interaction with F-box proteins (gray implies reduced activity; unpublished data) and consequently the poly-ubiquitination activity and proteasomal degradation of F-box proteins and/or substrates of the F-box proteins. Homology of elongin C of the E3^VB*C*^Ub-ligase with Skp1 of the E3^*S*CF^Ub-ligase is emphasized by the dotted purple line connecting their Ub-ligases.

Cells, whether free-living or residing within multicellular organisms, continuously monitor environmental O_2_ and integrate this information with other cues to regulate their metabolism, growth and development. Cytoplasmic prolyl 4-hydroxylases (P4Hs) are key O_2_ sensors in animals [1,2], owing to their ability to distribute the atoms of molecular O_2_ between the target Pro and the metabolite α-ketoglutarate. The transcriptional co-factor hypoxia inducible factor-α (HIFα) is a main target (Figure 1A), and hydroxylated HIFα is subject to polyubiquitination by the VHL (von Hippel-Lindau protein/cullin-2/elongin B/elongin C) type of E3 ubiquitin ligases leading to subsequent degradation in the 26S-proteasome [2]. Thus low O_2_ is thought to rapidly induce the expression of new genes appropriate to hypoxia. In contrast, a P4H in the social amoeba *Dictyostelium* and the human parasite *Toxoplasma gondii*, known as PhyA (previously referred to as P4H1), appears to solely hydroxylate Skp1 (Figure 1B), at Pro143 [3,4]. Hydroxylation does not affect Skp1 stability [5] but may regulate poly-ubiquitination activity of the SCF (Skp1/cullin-1/F-box) class of E3 ubiquitin ligases, of which Skp1 is an adaptor subunit [6,7]. The 4(*trans*)-hydroxyproline (Hyp) can then be sequentially modified by 5 sugars whose additions are catalyzed by 5 glycosyltransferase activities encoded by 3 genes [5,8,9]. Reverse genetic analyses demonstrated that hydroxylation and glycosylation of *Dictyostelium* Skp1 are essential for normal O_2_ regulation of development [10,11], and recent studies showed its importance for optimal growth of *Toxoplasma*[4].

**Figure 2:**
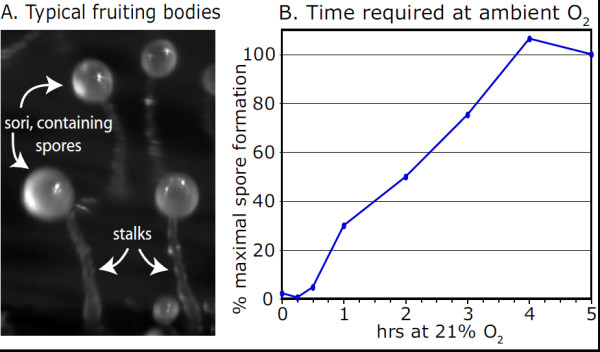
**O**_**2 **_**exposure required for culmination on filters.** (**A**) Morphology of typical strain Ax3 fruiting bodies formed at 24 h at an air-water interface on filters in ambient atmosphere (21% O_2_). Spores exclusively comprise the sori, which are supported aerially by cellular stalks. (**B**) Cells were allowed to develop for 12 h to the tipped aggregate stage before elongating to slugs, in an atmosphere of 5% O_2_. Filters were transferred to ambient atmosphere (21% O_2_) for the indicated period of time before return to 5% O_2_ to complete development. Culmination was quantitated by counting spores, which correlated with fruiting body formation (not shown). Results are typical of 2 independent trials.

*Dictyostelium* development is ultrasensitive to O_2_ making it a good model for understanding the mechanism of O_2_ sensing by other organisms that conserve the Skp1 modification pathway. Development is induced by starvation, which signals the normally solitary phagocytic amoebae to form a multicellular fruiting body, which consists of a cellular stalk that aerially supports thousands of spores for potential dispersal to other locations (see Figure 2A in Results) [12-14]. Initially, the amoebae chemotax together to form a multicellular aggregate, which polarizes in response to environmental cues and elongates into a migratory slug consisting of prestalk cells mostly at its anterior end and prespore cells in the remainder. The slug responds to environmental signals that direct its migration and regulate the slug-to-fruit switch– the process of culmination leading to formation of the fruiting body. Signals include light, low NH_3_, low moisture, higher temperature, and high O_2_ which, in the native environment of the soil, draw the subterranean slug to above ground where culmination is most productive [11,12,15-20]. In the laboratory, the process takes place over the course of 24 h after deposition of amoebae on moist agar or filter surfaces wetted with low salt buffers. Whereas amoebae grow and form slugs at an *air-water* interface in the presence of as little as 2.5% O_2_, ~10% is required for culmination [21], and slugs immersed in mineral oil require atmospheric hyperoxia to culminate [20]. Overexpression of Skp1 or absence of pathway activity drives the O_2_ requirement up to 18-21% (near ambient level), whereas decreased Skp1 or overexpression of PhyA drives the O_2_ requirement down to 5% or less [5,10,11]. These genetic manipulations also revealed effects on timing of slug formation and on sporulation. Together with studies on a Skp1 mutant lacking the modifiable Pro143 residue, and double mutants between Skp1 and pathway enzyme genes, the findings suggested that the Skp1 modification pathway mediates at least some O_2_ responses. However, O_2_ contingent modification of the steady state pool of Skp1 has not been demonstrated.

To address this issue, and to investigate the generality of O_2_ regulation of development, we turned to a previously described *submerged* development model in which terminal cell differentiation depends on high (≥70%) atmospheric O_2_[22,23]. The wider range of O_2_ concentrations presented to cells in this setting may facilitate analysis of the dependence of Skp1 hydroxylation on O_2_, and absence of the morphogenetic movements of culmination might reveal later developmental steps that are dependent on Skp1 and its modifications. In a static adaptation of the previous shaking cultures, we observed that terminal cell differentiation occurs in a novel radially symmetrical fashion in multicellular cyst-like structures. Under these conditions, we find that O_2_ is apparently rate-limiting for Skp1 hydroxylation, and that cyst formation and terminal spore differentiation that require high O_2_ also depend on normal levels of Skp1 and both its hydroxylation and glycosylation. This expands the role of Skp1 and its modifications in developmental regulation, and supports the model that O_2_ regulates its modification in cells.

## Methods

### *Dictyostelium* cell strains and growth

The normal *D. discoideum* strain Ax3 and its derivatives with the following genotypes were described previously: *phyA*^–^[3], *ecmA*::PhyA-myc/*phyA*^–^*, cotB*::PhyA-myc/*phyA*^–^[24], PKA(cat)/*phyA*^–^[24], *pgtA*^–^[8], PgtA-N/*pgtA*^–^[8], *agtA*^–^[25], *gmd*^–^[26], *ecmA*::Skp1A.1/Ax3, *ecmA*::Skp1A.2/Ax3, *cotB*::Skp1A.1/Ax3, *cotB*::Skp1A.3/Ax3, *cotB*::Skp1A3.H2/Ax3, *ecmA*::Skp1B.2/*phyA*^–^, *cotB*::Skp1A.2/*phyA*^*–*^, *cotB*::Skp1A.3/*phyA*^–^[10]. Note that the number before the decimal point represents alleles, and the number after represents clones that may vary in expression level. Cells were grown in shaking HL-5 axenic medium at 22°C [24], and collected before their density reached 0.8 × 10^7^/ml.

### Cell development

Cells were harvested by centrifugation (2000 *g* × 1 min) at 4°C, resuspended in PDF buffer (33 mM NaH_2_PO_4_, 10.6 mM Na_2_HPO_4_, 20 mM KCl, 6 mM MgSO_4_, pH 5.8), re-centrifuged and resuspended in PDF at 10^8^/ml, and deposited on 0.45 μm pore Millipore cellulose nitrate filters for standard development at an air-water interface [27]. For submerged development, washed cells were resuspended in PDF at 2 × 10^7^/ml and 1.4 ml was deposited into each well of a 6-well bacteriological or tissue culture plate (3 cm diameter wells). Plates were incubated for up to 72 h in a sealed plastic box, with inlet and outlet ports for gas flow, under room fluorescent lights at 22°C. The inlet valve was connected via a bubbling water humidifier to a compressed gas tank formulated with the indicated percentage of O_2_, with the balance made up of N_2_. Previously it was shown that inclusion of 1% CO_2_ did not affect the O_2_ dependence of culmination [24]. The outlet tube was connected to a Pasteur pipette held under water to monitor gas flow. Cultures were kept unstirred to prevent contact of cells or cell aggregates with the buffer surface, which led to polarization and/or floating fruiting bodies (data not shown). Volume and cell density were optimized for maximal spore differentiation at 100% O_2_ (data not shown). Alternate buffers, including KP (17 mM potassium phosphate, pH 6.5), or Agg buffer (0.01 M NaPO_4_, pH 6.0, 0.01 M KCl, 0.005 M MgCl_2_), yielded lower spore numbers.

Cell aggregates were visualized in a stereomicroscope using transmitted light, or using phase contrast illumination on an inverted microscope. For detection of cellulosic cell walls, samples were analyzed under epifluorescence illumination in the presence of 0.1% (v/v) Calcofluor White ST (American Cyanamid) in 10 mM potassium phosphate (pH 8.0), using DAPI-filters. Multiphoton confocal microscopy was performed at the OUHSC Imaging Laboratory on a Leica SP2 MP Confocal microscope.

For determining spore numbers, samples were supplemented with 0.2% NP-40, and spores were counted in a hemacytometer. Spores were identified based on their resistance to detergent, shape, refractility, and labeling with Calcofluor White ST or anti-spore coat Abs. Spore plating efficiency was determined by spreading an aliquot of detergent-treated spores on SM agar in association with *Klebsiella aerogenes*, and dividing the number of colonies by the counted number of input spores.

### Immunofluorescence

Spores were released from cysts by probe sonication in 0.2% NP-40 in KP, centrifuged at 13,000 g × 10 s, and resuspended in KP buffer. Spores were recovered from fruiting bodies on non-nutrient agar by slapping the inverted Petri plate on a counter and washing the spores from the lid, and processed in parallel. An aliquot was treated with 6 M urea, 1% (v/v) 2-mercaptoethanol in TBS (10 mM Tris–HCl, pH 7.4, 150 mM NaCl) for 3 min at 100°C prior to dilution in cold TBS and recovery by centrifugation. Spore suspensions (2 × 10^6^/50 μl) were deposited on glass slides onto which had been dried a 50-μl volume of 10 μg/ml poly-L-lysine in H_2_O. After 15 min, non-bound spores were removed by aspiration and washing with TBS. The monolayer was incubated in 4 mg/ml hemoglobin in TBS for 5 min, 1 μg/ml mAb 83.5 [28] in 4 mg/ml hemoglobin in TBS for 1 h, TBS (5 washes), 2 μg/ml Alexa 568-conjugated Rabbit anti-mouse IgG (Molecular Probes/Invitrogen) in 3% (w/v) bovine serum albumin in TBS, TBS (5 washes), and Vectashield mounting medium. Samples were analyzed through a 40× (N.A. 0.75) lens via the TRITC-channel of an Olympus epifluorescence microscope, and images were identically recorded using a SPOT Flex camera (Diagnostic Instruments) and processed using Photoshop CS3.

### Western blotting

Developing cells were collected by centrifugation at 2000 *g* × 1.5 min at 4°C and boiled for 2 min in Laemmli sample buffer containing 50 mM DTT. Low O_2_ samples were first supplemented with 2 mM sodium dithionite [5] to minimize possible hydroxylation during sample preparation. Whole cell lysates were resolved by SDS-PAGE on a 4-12% gradient gel (NuPAGE Novex, Invitrogen), and transferred to nitrocellulose membrane using an iBlot system (Invitrogen). Blots were probed with primary and fluorescent secondary Abs as described [10]. Blots were blocked in, and Abs were dissolved in, 5% non-fat dry milk in 20 mM Tris–HCl (pH 7.4), 150 mM NaCl, 0.02% NaN_3_, and Alexa 680 fluorescence was imaged using a Li-Cor Odyssey scanner. Prespore cell differentiation was probed using mAbs 5F5 and 83.5 [28], and Skp1 isoforms were detected using pAb UOK87 [5], pAb UOK85 [5], mAb 4H2 [29], mAb 1C9 [29], and mAb 4E1 [3]. Affinity-purified anti-actin was from Sigma Chemical Co.

Images were analyzed densitometrically using NIH Image J. mAb 4E1 was used in its linear response range [10] to obtain the fraction of Skp1 that was not modified. Initially, values for each upper and lower band were corrected for general background by subtraction of a blank intensity value obtained from the vicinity of the band of interest. Studies using pAb UOK87, which selectively recognizes unmodified Skp1, showed that 5% of Skp1 was unmodified at 100% O_2_ based on comparison with a *phyA*^–^ sample (not shown). The remaining density in the lower band of the 100% O_2_ sample is of uncertain identity but, since its level was observed to be proportionate to the level of the upper band (not shown), its value (as a fraction of the upper band) was subtracted from each sample in the O_2_ series. The fraction of unmodified Skp1 was determined by dividing the corrected intensity of the lower Skp1 band by the sum of the intensities of the lower and upper bands.

## Results

### Terminal differentiation at an air-water interface

*D. discoideum* amoebae develop to form fruiting bodies when dispersed in a low ionic strength buffer on a moist surface (Figure 2A). About 75% of the cells become aerial spores and the remainder form the structural stalk. At reduced O_2_ levels (2.5-10%), the slug intermediate continues to migrate on the surface without culminating [24]. When returned to the ambient O_2_ level (21%), culmination then occurs within about 5 h. To determine the minimal time required for exposure to ambient O_2_, slugs were exposed to 21% O_2_ for varying times before returning to low O_2_. Figure 2B shows that exposure to high O_2_ can be as brief as 1 h, though up to 4 h is required for maximal culmination based on spore counts. The requirement for high O_2_ appeared to be selective for *induction* of culmination, because terminal cell differentiation occurred normally even within the fruiting bodies formed after only 1 h of exposure to normoxia (data not shown). The effect of O_2_ appears to be mediated at least in part by prolyl 4-hydroxylation of Skp1, because elevated O_2_ levels are required by *phyA*^–^ and Skp1-overexpression strains, and lower O_2_ is required by PhyA overexpression and Skp1B^–^ cells [10,24]. To further explore the role of Skp1 modification in O_2_ sensing and the importance of culmination as the target of regulation, we turned to a previously described submerged development model [22,23,30], in which progress beyond the loose aggregate stage is strictly dependent on elevated atmospheric O_2_, and terminal differentiation bypasses the morphogenetic movements of culmination.

### Terminal differentiation in submerged cultures

**Figure 3:**
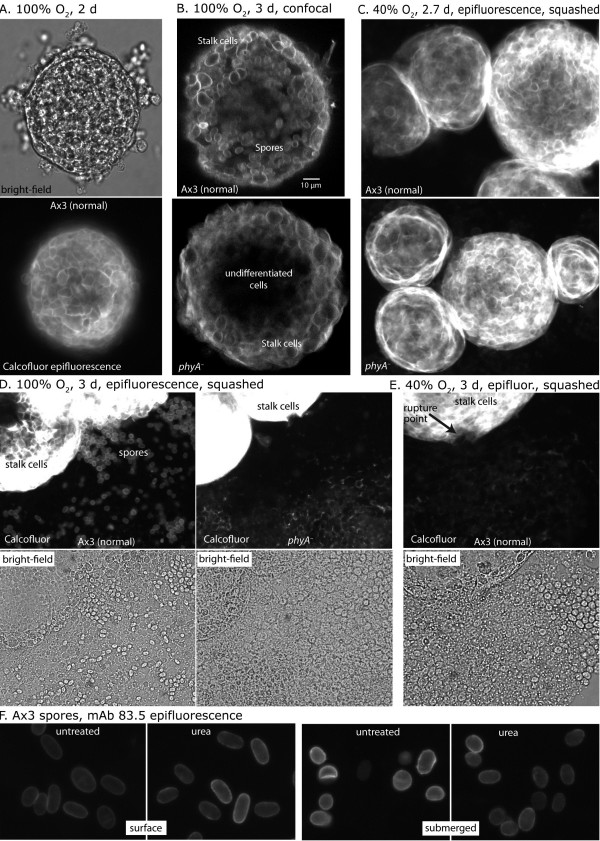
**Cell differentiation in submerged conditions.** Typical cyst-like structures formed by unstirred suspensions of strain Ax3 (normal) or *phyA*^–^ cells under the atmosphere of O_2_ percentage and for the duration indicated. (**A**) An Ax3 aggregate formed in 100% O_2_ was imaged by phase contrast (above), or epifluorescence microscopy in the presence of Calcofluor White ST (below) to reveal cell walls of terminally differentiated stalk cells at the aggregate surface. (**B**) Visualization of the interior of Ax3 and *phyA*^–^ aggregates using multiphoton confocal fluorescence microscopy in the presence of Calcofluor. (**C**) Aggregates of Ax3 or *phyA*^–^ cells formed under 40% O_2_ were squashed by applying vertical pressure to the cover slip, expelling some of the cellular contents resulting in wrinkling of the aggregate surface (evident as concentric folds appearing as rings). Cells were imaged for Calcofluor fluorescence. (**D**, **E**) Aggregates formed under 100% O_2_ (**D**) or 40% O_2_ (**E**) were similarly imaged, but exposure was adjusted to show fluorescence of expelled cells (absent in panel **C**), resulting in overexposure of the stalk cell-rich case. The point of emergence (rupture) of interior cells is indicated in panel **E**. (**F**) Spore coat formation. Spores from normal fruiting bodies developed at an air-water interface, and from submerged cultures maintained for 3 d under 70% O_2_, were compared by immunofluorescence labeling with mAb 83.5, which recognizes the fucose epitope predominantly on the spore coat proteins SP96 and SP75. Spores were labeled before or after extraction with urea/2-mercaptoethanol to permeabilize the coat. Control samples lacking mAb 83.5 exhibited only dim internal fluorescence (not shown).

**Figure 4:**
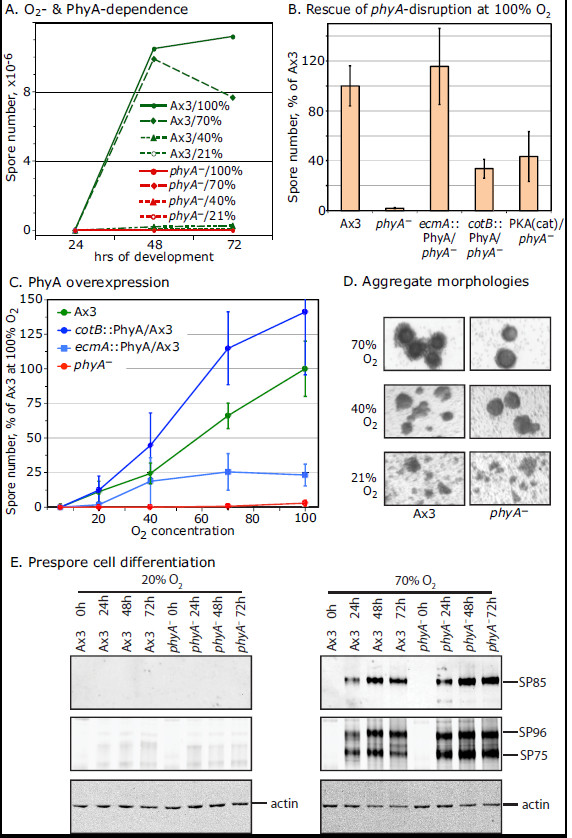
**Dependence of spore differentiation on PhyA.** (**A**) Spore differentiation of normal Ax3 (*phyA*^+^) and *phyA*^*–*^ cells in submerged conditions was quantitated as a function of time and [O_2_], by counting Calcofluor-positive spores in a hemacytometer. Data are from a single representative experiment. Note that data points for Ax3/21% O_2_ and the *phyA*^–^ strains overlap at the bottom. (**B**) Complementation of *phyA*^–^ cells by PhyA overexpressed under control of either the prestalk (*ecmA*) or prespore (*cotB*) cell specific promoter, and partial rescue of sporulation by expression of the catalytic domain of protein kinase A (PKAcat) under control of its own promoter. Spores were counted after incubation in 100% O_2_ for 72 h. Data represent the average and standard error of the mean (SEM) of 2–3 independent trials. (**C**) Effect of PhyA overexpression in normal (*phyA*^+^) cells as a function of O_2_. Spores were counted after 72 h. Data represent the average and SEM of 3–5 independent trials. (**D**) Typical cell cyst-like structures from samples in panel A were imaged using transmitted light. An increasing fraction of cells enter aggregates at higher O_2_, as inferred from fewer single cells in the background, and darker cores correlate with increased spore formation. (**E**) Cells developed for the indicated time were analyzed for the prespore cell differentiation markers SP85, and SP96 and SP75, based on Western blotting with mAb 5F5 and mAb 83.5, respectively.

When normal strain Ax3 cells were incubated at a similar density under a height of several mm of PDF buffer under room light illumination, rather than on a surface wetted with the same buffer, development proceeded only to the loose aggregate stage. However, when the atmosphere above the culture was maintained at 70 or 100% O_2_, the majority of cells formed tight spherical aggregates with diameters of 100–250 μm (Figure 3A) and optically dense cores (see Figure 4D below). These cell aggregates were uniformly bounded by Calcofluor-positive stalk cells, distinguished by their polygonal shapes due to cell expansion during terminal differentiation (Figure 3A). Confocal microscopy revealed that the stalk cells comprised a cortex surrounding an interior region of spore-like cells, based on their characteristic ellipsoid profiles, with an uneven boundary at the interface (Figure 3B). Note that Figures 3 and 4 also include comparative data on *phyA*^–^ cells (which do not modify Skp1), which will be described below. The interior cells could be liberated under pressure and consisted of a mixture of spores and undifferentiated (Calcofluor-negative) cells (Figure 3D). In contrast, the stalk cells remained associated with the deflated cyst-like structures. Maximal spore number was achieved by 2 d (Figure 4A), and ranged from 6 to 33% of the input cell number. These spores tended to be less elongated than their counterparts formed in fruiting body sori (see Figure 3F below), suggesting imperfect synchronization of spore coat assembly processes [28]. To test their authenticity, spores were released by probe sonication in a non-ionic detergent, which ruptured the cyst-like structures and lysed non-spore cells. Spores from cysts were on average slightly more brightly labeled than authentic spores isolated from fruiting bodies by immunofluorescence probing with mAb 83.5, which binds to the fucose epitope associated with the spore coat proteins SP96 and SP75 (Figure 3F). Surface labeling was retained even after boiling the spores in urea, indicating tight association of residual coat proteins with spore coat. To test spore function, equal numbers of spores prepared in this way were serially diluted in a clonal assay in association with *K. aerogenes* bacteria. The plating efficiency of cyst spores was 70%, similar to that of spores collected from fruiting bodies on filters, which was 66%. Thus, terminal cell differentiation occurred in radially symmetrical fashion in the absence of the normal morphogenetic movements of culmination. This contrasts with the slug-like elongated and linearly polarized aggregates formed when cells were agitated in high O_2_[22,23]. The radially polarized organization may result from a more uniform environment presented by the static setting in which polarizing gradients of O_2_ or NH_3_ fail to form.

Under 21% O_2_, stalk cells and spores were rarely observed in the less compacted aggregates that form under these conditions. When present they occurred as clusters or single cells (not shown). At 40% O_2_, larger aggregates were formed but they lacked dense cores observed at higher O_2_ levels. These cyst-like aggregates possessed a stalk cell cortex but their interior cells produced few spores, as visualized after squashing (Figures 3C,E). Though spores were not detected in this example, variable numbers were observed over the 5 independent trials as quantitated in Figure 4C. The variation suggests that 40% O_2_ is close to the threshold required for sporulation whose exact value is likely influenced by other factors, as observed for culmination [24]. To address the differentiation status of cells at the lower O_2_ levels, extracts were Western blotted for the spore coat precursor proteins SP85, SP96 and SP75 that are markers of prespore cell differentiation [31]. Whereas all 3 glycoproteins appeared in Ax3 cells by 24 h at 70% O_2_, negligible expression occurred at 20% after 3 d (Figure 4E). Thus increasing O_2_ levels were required for tight aggregate formation, terminal stalk cell differentiation, and differentiation of the interior prespore cells into spores. It is likely that metabolic O_2_ consumption results in intracyst hypoxia in these unstirred cultures which, in the submerged state, is not adequately replenished by O_2_ diffusion. The finding that elevated O_2_ tension in the atmosphere above the medium can rescue terminal differentiation indicates that O_2_ availability is the limiting factor for terminal cell differentiation in this setting. It is not evident whether the higher O_2_ level required for spore compared to stalk cell differentiation reflects a higher O_2_ threshold requirement for spore differentiation or lower O_2_ in the aggregate centers.

### Requirement of PhyA for sporulation in submerged conditions

A previously described mutant strain disrupted at its *phyA* locus [24] was analyzed to determine the involvement of Skp1 prolyl 4-hydroxylation in submerged development. *phyA*^–^ cells formed cyst-like structures at 40-100% O_2_ with outer layers of differentiated stalk cells, similar to the normal Ax3 strain (Figure 3C, D). However, interior cells failed to differentiate as spores, even after extended periods, as shown in the side-by-side comparisons in Figures 3B, D, 4A, and D. Instead, they remained as prespore cells, based on Western blot analysis showing abundant expression of the spore coat precursors (Figure 4E). Failure to sporulate was due to the PhyA deficiency, because *phyA*^*–*^ cells complemented with *ecmA*::*phyA* or *cotB*::*phyA*, which overexpress PhyA activity in prestalk or prespore cells respectively [24], were rescued at high O_2_ (Figure 4B). *ecmA*::*phyA/phyA*^*–*^ cells formed normal numbers of spores compared to Ax3, while *cotB*::*phyA/phyA*^*–*^ only partially rescued spore formation to about 30% of Ax3 levels. The difference suggests that prestalk cells may be important in mediating the role of PhyA in sporulation, consistent with evidence for a role of prestalk cells in processing or mediating sporulation signals during normal culmination [32-34]. While overexpression in prespore cells (*cotB* promoter) was also partially effective, the possibility that PhyA signals autonomously in prespore cells is not proved because on filters, *cotB*::PhyA^oe^ cells tend to migrate to the tip in chimeras with normal cells [24]. Successful complementation from these developmental promoters confirmed that cells had differentiated into prestalk and prespore cells in the absence of PhyA, and showed that PhyA is required only after their appearance. Since spore formation selectively depended on high O_2_ and the threshold for spore (but not stalk cell) differentiation was specifically affected by the absence of PhyA, PhyA activity appears to have a novel function in mediating O_2_ regulation of spore differentiation.

Since overexpression of PhyA in a *phyA*^+^ (wild-type) background reduces the O_2_ level required for culmination on filters [24], the effect of PhyA overexpression on sporulation was investigated. As shown in Figure 4C, modestly increased sporulation was observed at 70% O_2_ when PhyA was overexpressed in prespore cells. However, overexpression in prestalk cells inhibited sporulation, without affecting cyst formation *per se*. As noted above, PhyA overexpression under the *ecmA* promoter in a *phyA*^–^ background rescued sporulation better than under the *cotB* promoter, so the inhibitory effect of overexpression in *phyA*^+^ cells appears to be depend on a complex interplay between relative levels of expression in the different cell types rather than a cell autonomous effect on prestalk cells.

### Skp1 modification is O_2_ dependent

**Figure 5:**
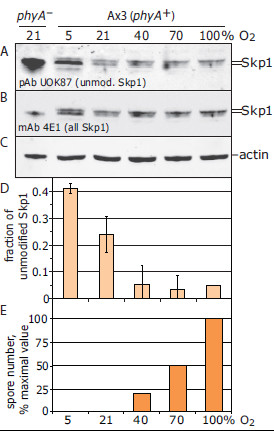
**O**_**2 **_**is rate limiting for Skp1 hydroxylation.** The modification status of Skp1was analyzed as a function of O_2_ level. Whole cell extracts (Ax3 or *phyA*^–^) harvested after 24 h of submerged development were subjected to SDS-PAGE and Western blotting. Blots were sequentially probed with (**A**) pAb UOK87, which preferentially (but not exclusively) recognizes unmodified Skp1 [5], (**B**) mAb 4E1, which recognizes all Skp1 isoforms, and (**C**) anti-actin, as a loading control. Skp1 migrated predominantly as 2 bands: an upper glycosylated band, and a closely-spaced lower non-glycosylated band (preferentially labeled by pAb UOK87) that was partially contaminated by glycosylated Skp1. (**D**) The levels of glycosylated and unmodified Skp1 were quantitated by densitometry of mAb 4E1-labeled blots as described in Methods, and the fraction of total Skp1 that is unmodified is plotted as a function of O_2_ level in the atmosphere. Data are from 5 independent experiments and are reported ± SEM. (**E**) The number of spores formed after 3 d for the trial shown for panels **AC** is shown.

To determine if Skp1 hydroxylation is affected by O_2_ availability, its modification status was assessed by Western blotting with pan- and isoform-specific Abs. Extensive analysis of soluble Skp1 from growing and developing cells shows that ≥90% of the steady state pool is homogenously modified by the pentasaccharide, and ~5% exists in unmodified form. Fully modified and unmodified Skp1 migrate as a doublet in SDS-PAGE and, though the resolution of the doublet is compromised when whole cell extracts are analyzed, isoform-specific Abs indicate that total cell Skp1 is modified to a similar extent [5,10]. After 1 d of submerged development, total Skp1 from 40, 70 or 100% O_2_ cells migrated mainly as the upper band using mAb 4E1 that recognizes all Skp1 isoforms (Figure 5B). In comparison, 5% O_2_ cells accumulated substantial Skp1 in the position of the lower band. This band corresponds to unmodified Skp1 based on reactivity with pAb UOK87 (Figure 5A). UOK87 preferentially binds unmodified Skp1 but exhibits weak reactivity with all Skp1 isoforms, so the upper band is also labeled. The lower band was not recognized by pAb UOK85 or mAb 1C9, which are specific for HO-Skp1 and GlcNAc-O-Skp1, respectively (data not shown). Quantitation of 5 independent samples indicated that the fraction of unmodified Skp1 decreased from 41% at 5% O_2_, to 24% at 21% O_2_ and 5% at 40% and higher levels (Figure 5D). Similar results were observed after 2 d of development except that the fraction of unmodified Skp1 at the lower O_2_ levels was slightly increased (data not shown). Since Skp1 turns over slowly with a half-life of 12–18 h during filter development [5,35], it is likely that the appearance of non-glycosylated Skp1 was the result of new synthesis and that at 5 and 21%, O_2_ is rate limiting for Skp1 hydroxylation. As shown in panel E, sporulation depended on higher levels of O_2_ than required to hydroxylate Skp1. Although 40% O_2_ was sufficient to ensure that the steady-state pool of Skp1 was maximally hydroxylated within the sensitivity of our assay, a delay in hydroxylation of nascent Skp1 of several hrs would have escaped our detection, and may be biologically relevant for sporulation (see Discussion).

### Role of glycosylation in submerged development

**Figure 6:**
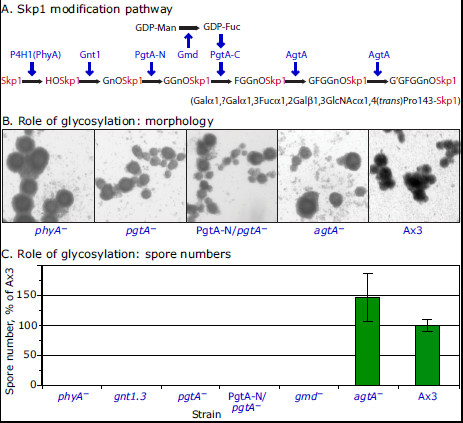
**Dependence of spore differentiation on Skp1 glycosylation.** (**A**) Schematic of the Skp1 modification pathway. Enzymes are in indicated by gene names; G= Gal; F= Fuc; Gn= GlcNAc. (**B**) Normal cells (Ax3) and modification pathway mutants were developed at 100% O_2_ in submerged conditions for 72 h. All strains formed similar tight aggregates, except that Ax3 aggregates exhibited dense cores; *agtA*^–^ aggregates formed few spores in this trial. (**C**) Spore numbers were determined as in Figure 4. Average values ± SEM from 5 independent trials are shown. The wide error bar for *agtA*^–^ cells results from a range of outcomes from near zero to more than Ax3.

Disruption of *phyA* also blocks hydroxylation-dependent glycosylation of Skp1, which occurs according to the scheme in Figure 6A. To investigate the role of glycosylation *per se*, *gnt1.3*, *pgtA*^*–*^, *gmd*^*–*^, *pgtA-N/pgtA*^*–*^, and *agtA*^–^ cells, which accumulate Skp1 with zero, one, two, two, or three sugars respectively [5,8,26] on account of enzyme gene disruptions, were analyzed. The strains expressing up to two sugars formed cyst-like structures which, however, failed to acquire dense-cores or induce spore formation, like *phyA*^*–*^ cells (Figure 6B, C). In contrast, *agtA*^*–*^ cells, which accumulate the trisaccharide form of Skp1 [25], were inconsistent in spore formation with numbers ranging from essentially zero to more than Ax3. Thus although the final two sugars were not always required for sporulation, their absence appears to make sporulation vulnerable to an unknown variable. Potential sources of variation include NH_3_ and light, which were previously shown to influence the O_2_ threshold for culmination on filters [24], and conditioned medium factors previously detected during submerged development [30]. Taken together, the results suggest that the role of hydroxylation may be simply to support glycosylation. This contrasts with culmination, in which hydroxylation alone partially rescues the normal O_2_ requirement of *phyA*^*–*^ cells [5], an effect that is reversed by the action of PgtA in the absence of AgtA [9].

### Role of Skp1 and its modifications in submerged development

**Figure 7:**
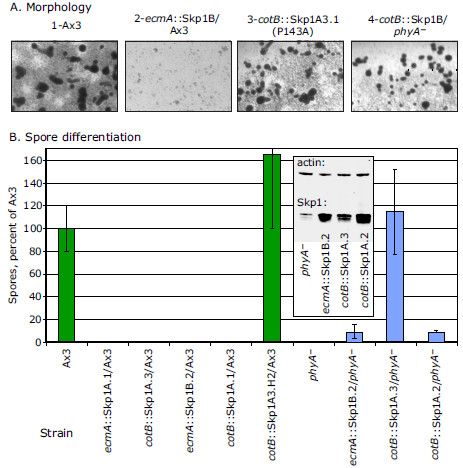
**Dependence on Skp1 expression level.** Strains overexpressing Skp1A or Skp1B under control of either the *ecmA* or *cotB* promoter, in either a normal or *phyA*^*–*^ background, were developed in submerged conditions beneath 100% O_2_ for 72 h. Typical results from selected strains are shown. (**A**) All tested strains formed tight aggregates except those overexpressing wild-type Skp1 in a *phyA*^*+*^ background, which remained as small, loose aggregates. (**B**) Spore numbers were counted and normalized to Ax3 spore counts in the same trial. Average values ± SEM from 2–3 independent trials are reported. Inset shows a Western blot for Skp1, showing its level of overexpression in the *phyA*^–^ strains.

The role of Skp1 itself was investigated by overexpression in different genetic backgrounds. Native Skp1 sequences were employed because a previous study showed that N- or C-terminal peptide tags interfere with its hydroxylation and activity in cells [10]. Overexpression of Skp1B under the *ecmA* (prestalk) promoter inhibited tight aggregate formation even at 100% O_2_ (Figure 7A-2). No spores (Figure 7B) and few stalk cells (not shown) were observed, confirming inability to progress past this early stage. Similar results were observed with a strain overexpressing the closely related isoform Skp1A (which differs by a single amino acid), or when either Skp1 was expressed under control of the *cotB* promoter (Figure 7B). However, overexpressing mutant Skp1A3(P143A), which cannot be modified, did not interfere with aggregation (Figure 7), and wild-type Skp1 overexpression failed to inhibit cyst formation in the absence of PhyA (Figure 7A-4). These strains did not form cyst-like structures or spores at lower O_2_ levels (data not shown), implying that high O_2_ also provides an additional, possibly metabolic, function important for development. The opposing effects of Skp1 overexpression and blocking its modification suggests that modification stimulates Skp1 activity, which can be modeled as breakdown (by a specific E3^SCF^ubiqutin ligase) of a hypothetical *activator* of cyst formation.

In comparison, the requirement of Skp1 glycosylation for sporulation suggests that for this later developmental step, Skp1 contributes to the breakdown of a hypothetical *inhibitor* of sporulation. Without modification, Skp1 is not activated and the inhibitor accumulates. However, overexpression of Skp1 in the *phyA*^–^ background (thereby bypassing the block to cyst formation) allows sporulation, which can be interpreted as providing additional activity to compensate for lack of activation by modification (Figure 7B, blue bars and inset; data not shown). Similar effects were observed irrespective of the promoter used, or whether wild-type Skp1A or B, or mutant Skp1, was overexpressed (data not shown). However, overexpression of Skp1 at very high levels did not rescue sporulation in *phyA*^–^ cells as well, which might reflect a dominant negative effect toward SCF-complex formation. Separate effects on activators and inhibitors may depend on involvement of distinct F-box proteins.

## Discussion

Three novel observations regarding development under submerged conditions are presented here: i) In the presence of high O_2_ and absence of stirring, cell differentiation occurs in a radially symmetrical rather than the typical linearly polarized pattern. With their outer husk-like cortex and interior germinative cells, these structures have the organization of multicellular cysts as occur in animal tissues. The cyst-like structures are distinct from other terminal states formed by *Dictyostelium*, including the dormant unicellular microcyst and the multinucleated macrocyst [36]. Although conditions leading to the formation of cyst-like structures are not known to occur naturally, its O_2_ dependence is likely to be relevant to interpreting O_2_ signaling in normoxia as outlined below. ii) Skp1 hydroxylation is limited by O_2_ availability. iii) Certain developmental transitions that occur during submerged development, including tight aggregate formation and terminal spore differentiation, critically rely on hydroxylation and glycosylation of Skp1. Together, these findings reinforce a role for environmental O_2_ for influencing polarity and key developmental transitions, and strongly implicate the Skp1 modification pathway in decoding the O_2_ signal.

### Significance of O_2_ for control of polarity and terminal differentiation

**Figure 8:**
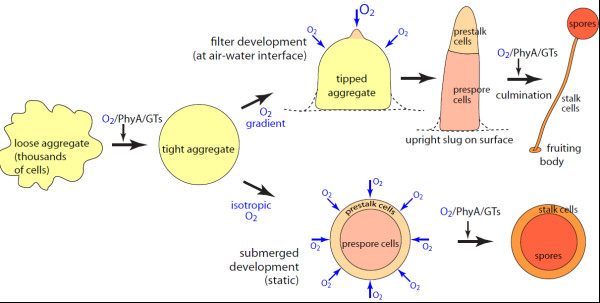
**Model for O**_**2 **_**regulation of development and dependence on the Skp1 modification pathway.** Cells form loose aggregates that condense into tight aggregates by a Skp1 associated, O_2_ dependent mechanism based on submerged development studies (Figures 4D, 7A-2). At a conventional air-water interface (upper track, depicted by dashed line), the exposed apical surface supports maximal O_2_ transport which is proposed to induce tip formation whose smaller radius of curvature encourages even more O_2_ transport, stabilizing the tip as an organizer and the zone where cells differentiate as prestalk cells. The Skp1 modification pathway, under regulation of O_2_ and other factors, regulates culmination and sporulation at the air-water interface. Under submerged conditions (lower track), metabolism consumes O_2_, which becomes depleted owing to slow diffusion in the unstirred cultures. In this isotropic and hypoxic environment, all (not just apical) surface cells become prestalk cells and, in the absence of a polarizing O_2_ gradient, cells differentiate *in situ* and those that sense the lowest O_2_ level (which occurs in the center) become spores. O_2_ action may be complementary to NH_3_, a volatile inhibitor that is generated during development and is preferentially lost from the same surfaces by diffusion (see Discussion).

Formation of the novel cyst-like structures is compared to normal development at an air-water interface as a backdrop to interpreting the role of Skp1 modification in O_2_ signaling. During normal development at an air-water interface, the tip emerges at the apex of the hemispherical aggregate and exerts a dominant role in controlling elongation into a slug, slug migration, internal cell dynamics, and the induction and orchestration of the morphogenetic movements of culmination [13,14,16,20,37]. The tip, composed of prestalk-type cells [38], senses environmental signals, including O_2_ potentially, and relays the information to the other slug cells to follow suit (Figure 8, upper track). In previous submerged development studies, cells were shaken under an atmosphere of high O_2_ and the aggregates elongated into slug-like structures in which prestalk and prespore cells segregated toward opposite ends and terminally differentiated *in situ*[22,30,31,39]. In the absence of stirring as described here, cell aggregates instead become spherical cysts in which internal prespore and spore cells are surrounded by stalk cells. These findings suggest that O_2_ contributes to patterning and terminal differentiation, as follows (Figure 8, lower track). Given that O_2_ is metabolically depleted in the aggregate center, a gradient of O_2_ occurs with the highest levels at the aggregate surface [39] where the O_2_ level is expected to be uniform all they way around. Based on studies in capillaries [40] and in agar immobilized aggregates [39], it is likely that the higher O_2_ level at the aggregate surface attracts spontaneously differentiated prestalk cells and triggers their terminal differentiation. This is consistent with the transient existence of a monolayer of prestalk-like cells that has been observed at the slug surface [41]. Higher than ambient O_2_ might be required as a consequence of the submerged condition in which replacement diffusion of O_2_ lags behind metabolic consumption. In the absence of orienting signals in this isotropic setting, the aggregate remains radially-polarized. However, at the air-water interface, tip formation initiates at the apex of the aggregate owing to highest O_2_ accessibility, which becomes stabilized as its smaller radius of surface curvature ensures greatest gas exchange with the underlying cells. The interior prespore cells, experiencing relative hypoxia owing to metabolic consumption of O_2_, might not normally differentiate until culmination permits aerial exposure to atmospheric O_2_ levels or modulates metabolites that regulate PhyA and the glycosyltransferases. The idea that hypoxic niches regulate cell differentiation has precedent in studies on animal stem cells and maize germ cells [42,43].

NH_3_, a volatile metabolite released during the massive breakdown of protein during development [44], has also been implicated as a polarity factor and inhibits the slug-to-fruit switch [16]. Since NH_3_ is expected to diffuse away most at the same surfaces that O_2_ is expected to diffuse in, the two compounds may play complementary inhibitory and activating roles that tune developmental decisions. Thus, while hypoxic or *phyA*^–^ preculminants may still form tips at the air-water interface [24] due to the NH_3_ effect, the spherical shapes assumed by *phyA*^–^ slugs after long periods of migration [5] might reflect eventual depletion of the NH_3_ signal as protein is finally consumed. The isotropic environment during static submerged development may thwart formation of orienting NH_3_ as well thereby resulting in radial polarization, and high NH_3_ in the interior is expected to promote sporulation [45]. Since NH_3_-signaling is mediated in part by NH_3_-transporter/sensors [16,17], investigation of genetic interactions with *phyA* may allow understanding of the interplay with Skp1 modification.

### Role of Skp1 prolyl hydroxylation in tight aggregate formation

Tight aggregate formation depended on an elevated O_2_ level of ≥40%, but this was inhibited when Skp1 (either isoform) was overexpressed under either developmental promoter (Figure 7A). This correlates with the 7-hr delay of the loose-to-tight aggregate transition of these overexpression strains at the air-water interface [10]. Interestingly, inhibition of tight aggregate formation was partially relieved when Skp1 was overexpressed in a *phyA*-mutant background, which also relieved the delay on filters. Consistent with a requirement for modification, overexpression of Skp1A3(P143A), which cannot be hydroxylated, is not inhibitory (Figure 7A, B). The opposing effects of Skp1 overexpression and inhibiting its modification are consistent with a model in which modification activates Skp1 and its role in polyubiquitination and breakdown of a hypothetical activator of cyst formation.

### Role of Skp1 prolyl hydroxylation and glycosylation in sporulation

A second function of the pathway was revealed by the essentially complete failure of the interior prespore cells to differentiate in the *phyA*^–^ strain, whereas stalk cell differentiation was qualitatively unaffected (Figures 3, 4). The blockade was overcome when PhyA was overexpressed in prestalk and to a lesser extent prespore cells (Figure 4B), so control by O_2_ may be mediated via prestalk cells. This is consistent with evidence that prestalk cells can regulate sporulation via processing of spore differentiation factor-1 and −2 [33,34]. However, the role of PhyA appears complex because overexpression in prestalk cells in the *phyA*^+^ (wild-type) background inhibited sporulation, as if relative levels of O_2_ signaling between cell types could be important. The blockade was also partially overcome when PKA activity was promoted by overexpression of its catalytic domain under its own promoter (Figure 4B). Since PKA expression in prespore cells was previously shown to be sufficient for activating sporulation [46], PhyA may signal upstream of PKA as suggested for its role in culmination on filters [24].

Hydroxylated Skp1 is a substrate for Gnt1 that in turn generates a substrate for PgtA, and then AgtA, resulting in formation of the pentasaccharide on Hyp143 (Figure 6A). Mutants lacking enzymes to extend to the trisaccharide state were also unable to sporulate at high O_2_ (Figures 6B,C), suggesting that hydroxylation supports extension of the glycan chain to three or more sugars to trigger sporulation. Though the preceding culmination step (on filters) exhibited more modest dependence on addition of the first two sugars (at lower O_2_ levels) [5], the more dramatic difference in the static submerged model may simply result from failure to achieve a critical threshold of O_2_ in the cyst interior. The greater difference was in the role of AgtA, whose contribution was almost as important for culmination as PhyA [9] but was unnecessary for submerged sporulation. Thus the role of AgtA appears to be specialized for culmination compared to sporulation.

The requirement of PhyA for sporulation was partially overcome by overexpression of Skp1 (Figure 7). This suggests that PhyA action normally promotes Skp1 activity, and its absence can be bypassed by excess Skp1. A related effect was observed on filter development, where Skp1 overexpression inhibited sporulation at high O_2_ levels that allowed culmination, but removal of PhyA blocked inhibition [10], indicating that PhyA tunes Skp1 activity. This is consistent with activation of Skp1 poly-ubiquitination activity toward an inhibitor. In comparison, the effect of Skp1 modification on culmination implied *inhibition* of Skp1 breakdown activity toward a hypothetical activator [10,11], and the effects on cyst formation (assessed morphologically) above suggested *activation* of breakdown activity toward an activator. These disparate effects are consistent with what is known about the SCF family of E3 ubiquitin-ligases, which polyubiquitinate different substrates depending on which F-box protein is present. Furthermore, these Ub-ligases can have opposite effects via auto-polyubiquitination of the F-box protein itself, which results in protection of the substrate receptor [6,7]. Conceivably, Skp1 modification may selectively affect these different activities.

### O_2_ is limiting for Skp1 hydroxylation in submerged culture and mechanistic implications

In submerged development, substantial levels of unmodified Skp1 (Figure 5D) accumulated at 5% and 21% O_2_. Since i) there is no evidence for enzymatic reversal of hydroxylation or glycosylation, ii) the level of Skp1 was similar at different O_2_ levels, and iii) Skp1 turns over with a half-life of 12–18 h [5], it is likely that appearance of unmodified Skp1 was due to failure to hydroxylate nascent Skp1. Since the total Skp1 pool becomes 95% hydroxylated at ≥40% O_2_ (Figure 5D), O_2_ is likely rate-limiting for Skp1 prolyl hydroxylation. This is consistent with co-expression evidence that PhyA is rate limiting for Skp1 hydroxylation [10]. Since sporulation is minimal at 40% O_2_ even though the steady-state pool of Skp1 appears fully modified, it may be that O_2_ and PhyA have additional or alternative mechanisms for controlling sporulation. However, it should also be considered that a several hour delay in the hydroxylation of nascent Skp1, which might be most important for partnering with nascent F-box proteins, would have escaped detection against the background of total Skp1 using our methods.

Since the Skp1/F-box protein complex is characterized by a high affinity [29] that is increased by hydroxylation as suggested in Figure 1B (M.O. Sheikh and C.M. West, unpublished data), we propose that even transient accumulation of unmodified Skp1 will influence the spectrum of complexes with one or more of the ~38 predicted F-box proteins that are strongly up and/or down-regulated at various times during development based on RNAseq data [47] (unpublished studies). This in turn may affect the timing of developmental transitions via effects on the stability of F-box proteins and hypothetical F-box protein substrates (activators and inhibitors) that normally control aggregation, slug formation, culmination and sporulation [e.g., [48]. Figure 2B shows that O_2_ exposure of 1–3 h can rescue culmination of hypoxic slugs, consistent with a transient role that might correlate with expression of a specific F-box protein. Current studies are focused on how Skp1 modification influences E3^SCF^ubiquitin-ligase assembly and activity.

These findings in social amoebae may be pertinent to numerous protist groups, including other amoebae (e.g., *Acanthamoeba*), plant pathogens (*Phytophthora*), diatoms (brown algae), green algae (*Chlamydomonas*), ciliates (*Tetrahymena*), and apicomplexans including *Toxoplasma*, whose O_2_ dependence have been little studied but whose genomes harbor Skp1 modification pathway-like genes [11]. For example, recent studies [4] showed that the related Skp1 modification pathway supports growth of *Toxoplasma* in cultured fibroblasts especially at low O_2_.

## Conclusions

In an isotropic submerged environment under high O_2_, starved *Dictyostelium* cells form cyst-like structures in which terminal differentiation occurs in a radially symmetrical pattern consisting of external stalk cells and internal spores. Low O_2_ is rate-limiting for the hydroxylation and subsequent glycosylation of Skp1, which correlates qualitatively with inhibition of spore differentiation. Genetic perturbations indicate the importance of Skp1 hydroxylation and glycosylation for activating Skp1 activity in regulating cyst formation and sporulation, in addition to previous evidence for its inhibition in regulating culmination at an air-water interface. The findings support a model in which environmental control of Skp1 modification differentially influences sequential developmental transitions via polyubiquitination and degradation of F-box proteins and their respective regulatory factor substrates.

## Abbreviations

Hyp: (*4R*,*2S*)-hydroxyproline (aka 4(*trans*)-hydroxy-L-proline); mAb: Monoclonal antibody; pAb: Polyclonal antibody; PhyA: Prolyl 4-hydroxylase-1 from *D. discoideum*; PKA: Protein kinase A; SCF: E3 ubiquitin ligase sub-complex consisting of Skp1, a cullin-1, an F-box protein, and Rbx1; SEM: Standard error of the mean; Ub: Ubiquitin.

## Competing interests

The authors declare that they have no competing interests.

## Authors’ contributions

RSG performed initial experimentation including optimizations. ZAW broadened the scope of the study to include the complete mutant panel, and wrote the first draft. YX confirmed all the findings and conducted most of the molecular characterizations. CMW trained the students, coordinated the study, conducted some experiments, and wrote the manuscript which was approved by all authors.
